# Therapeutic potential of growth differentiation factors in the treatment of degenerative disc diseases

**DOI:** 10.1002/jsp2.1045

**Published:** 2019-03-08

**Authors:** Tom Hodgkinson, Bojiang Shen, Ashish Diwan, Judith A. Hoyland, Stephen M. Richardson

**Affiliations:** ^1^ Division of Cell Matrix Biology and Regenerative Medicine, School of Biological Sciences, Faculty of Biology, Medicine and Health, University of Manchester Manchester Academic Health Sciences Centre Manchester UK; ^2^ Centre for the Cellular Microenvironment, Institute of Molecular, Cell and Systems Biology, College of Medical, Veterinary and Life Sciences University of Glasgow Glasgow UK; ^3^ St. George Clinical School University of New South Wales Sydney New South Wales Australia; ^4^ NIHR Manchester Biomedical Research Centre, Manchester University Foundation Trust Manchester Academic Health Sciences Centre Manchester UK

**Keywords:** annulus fibrosus, bone morphogenetic protein, cartilage derived morphogenetic protein (CDMP), growth differentiation factor (GDF), intervertebral disc degeneration, nucleus pulposus, mesenchymal stem cell

## Abstract

Intervertebral disc (IVD) degeneration is a major contributing factor to chronic low back pain and disability, leading to imbalance between anabolic and catabolic processes, altered extracellular matrix composition, loss of tissue hydration, inflammation, and impaired mechanical functionality. Current treatments aim to manage symptoms rather than treat underlying pathology. Therefore, IVD degeneration is a target for regenerative medicine strategies. Research has focused on understanding the molecular process of degeneration and the identification of various factors that may have the ability to halt and even reverse the degenerative process. One such family of growth factors, the growth differentiation factor (GDF) family, have shown particular promise for disc regeneration in in vitro and in vivo models of IVD degeneration. This review outlines our current understanding of IVD degeneration, and in this context, aims to discuss recent advancements in the use of GDF family members as anabolic factors for disc regeneration. An increasing body of evidence indicates that GDF family members are central to IVD homeostatic processes and are able to upregulate healthy nucleus pulposus cell marker genes in degenerative cells, induce mesenchymal stem cells to differentiate into nucleus pulposus cells and even act as chemotactic signals mobilizing resident cell populations during disc injury repair. The understanding of GDF signaling and its interplay with inflammatory and catabolic processes may be critical for the future development of effective IVD regeneration therapies.

## 1 INTRODUCTION

Low back pain places a significant socioeconomic burden on society, with ~632 million people affected globally.^1^ Approximately, 84% of people will experience low back pain during their lifetime, leading to associated annual expenditure of £12 billion in the United Kingdom, with similar costs reported in other developed countries (eg, $85.9 billion in the United States and €16.5‐50 billion in Germany).^2^, ^3^ This cost arises from direct medical expenses, work absences and wage compensation^1^, ^4^, ^5^ and surpasses that of many other causes of disability, including arthritis.^6^, ^7^ The incidence of low back pain and associated cost are rising dramatically as the current global demographic shifts toward an increasingly aged population.^8^

Although low back pain is multifactorial and complex in etiology, intervertebral disc (IVD) degeneration has long been identified as a major underlying cause.^9^, ^10^, ^11^ The IVDs are fibrocartilaginous tissues positioned between the vertebrae, contributing to about one‐third of total spinal length.^12^ Functionally IVDs are crucial structural components responsible for conferring mechanical strength and flexibility to the vertebral column.^13^, ^14^ IVD degeneration is thought to arise from cell driven changes to the extracellular matrix (ECM) of the central portion of the disc, the nucleus pulposus (NP), which results in mechanical failure of the NP and annulus fibrosus (AF; a collagenous tissue circumferentially enclosing the NP), progressive AF fissure formation and eventual NP herniation.^15^ This process is concurrent with an in‐growth of blood vessels and nociceptive nerve fibers into the inflamed disc, facilitating immune cell infiltration and increasing associated pain.^16^, ^17^ The progressive obstruction of the IVDs ability to absorb and disperse spinal loads through the motion segment (the structural unit comprising the IVD, facet joints and adjacent vertebral bodies) in degeneration is secondarily linked with facet joint arthritis, spur/osteophyte formation, and vertebral body deformation. These have been associated with degenerative spinal conditions such as spinal cord stenosis, spondylolysthesis, degenerative scoliosis, and other painful pathologies resulting from nerve compression, such as sciatica.^9^, ^18^ IVD degeneration can be exacerbated by excessive manual labour, underlying genetic factors, and the aging process.^6^

As a natural phenomenon of aging, some aspects of IVD degeneration may be difficult to prevent.^10^, ^19^ Indeed, the majority of adults over 30 years show some form of structural IVD degeneration without any accompanying symptoms or pain.^6^ This makes diagnosis and effective early intervention in cases of emerging pathogenic degeneration a priority. Current treatment options are limited and provide predominately symptomatic relief without addressing the underlying pathology. These can be broadly grouped into, first, conservative treatments, ranging from painkillers and anti‐inflammatory medication to physiotherapy, and second, surgical interventional. Surgery is utilized as a last resort, with procedures such as discectomy and spinal fusion costly to perform and resulting frequently in suboptimal healing outcomes and recurrence.

Therefore, there is great demand for a biological treatment aimed at restoring IVD homeostasis and regenerating damaged tissue. Of importance to such strategies is the restoration of both structure and function of the NP and AF tissues. To this end, biological therapies have shown promise in preclinical studies. These could include cellular and acellular therapies delivered with and without instructive biomaterials and in conjunction with bioactive molecules or growth factors (see^20^ for recent in‐depth review). One such family of factors, growth differentiation factors (GDFs), appear to be an exciting prospect due to their crucial role in chondrogenesis (including differentiation to NP cells, namely, discogenesis) and cartilaginous tissue homeostasis.^21^, ^22^, ^23^, ^24^ As such, the focus of this review is directed on the continuing development of regenerative strategies for IVD repair employing GDF family members and the potential therapeutic role of GDF6.

## 2 IVD STRUCTURE, FUNCTION AND DEGENERATION

The IVD can be described as three distinct regions—the NP, AF, and cartilaginous endplates (CEPs)—making up the largest avascular structure in the human body. The properties and functionality of the IVD are dependent on the specific microstructures of its component tissue regions, which in turn are produced by distinct cell populations.

The NP is characterized by an ECM that is rich in anionic proteoglycans (PGs), predominately aggrecan. These are arranged within an irregular type II collagen lattice and are present at a ratio of 27:1 (aggrecan: collagen).^18^, ^25^ Additional matrix components consist of small amounts of other collagen types (I, VI, IX, and XI) along with other hydrophilic aggregating PGs, such as versican, and nonaggregating small leucine‐rich PGs such as biglycan, decorin, fibromodulin, keratocan, and lumican.^26^, ^27^, ^28^, ^29^, ^30^ The high density of negatively charged PG molecules draws in and retains water allowing the NP to resist compressive loads.

Circumferentially, the NP is enclosed by the AF, a ligamentous structure composed of highly organized collagen fibers arranged in concentric lamellae, with superior and inferior fiber ends rigidly anchored in the CEPs.^31^ The AF is composed of more than ⅔ collagen and unlike the NP has a small PG component.^32^, ^33^ More than 95% of the collagen in the outer AF is type I, decreasing in an almost linear fashion to less than 5% with proximity to the NP. An opposing gradient exists for type II collagen going from the center of the NP to the outer AF.^34^ In successive lamellae, type I collagen fibers are obliquely oriented at angles of approximately 62 to 47 to the spinal axis, preventing IVD deformation under load.^35^

The CEPs physically confine the NP and AF to their anatomical boundaries and act as semipermeable barriers, supporting nutrient and fluid exchange. The anchorage of AF fibers to the CEPs superiorly and inferiorly is also critical to the integrity of the motion segment and is strengthened by fiber bundle splitting to increase force distribution.^36^, ^37^, ^38^, ^39^

During IVD degeneration, type II collagen synthesis by the NP cells is gradually replaced by type I collagen, while PG synthesis is decreased resulting in the boundaries between the NP and AF becoming less distinct. The high PG content is central to healthy NP function, and the ratio of PG‐to‐collagen is one of the defining features sets NP cells apart from chondrocytes. This is important to note for regeneration of the NP, where implantation of chondrocytes rather than NP cells may result in a cartilage‐like matrix with insufficient PG concentrations.^40^ Deficient water‐retention by the NP results in a decrease in disc height, which in turn leads to a loss of ability to uniformly distribute compressive forces to the AF, creating areas of high pressure.

The cells of the NP are highly specialized, now recognized through detailed transcription profiling and murine cell tracing studies to be developmentally and phenotypically distinct from chondrocytes.^41^, ^42^, ^43^, ^44^, ^45^, ^46^, ^47^, ^48^, ^49^, ^50^ The Spine Research Interest Group of the Orthopedic Research Society recently defined the NP phenotype to include stabilized expression of hypoxia inducible factor HIF‐1α, the glucose transporter glut‐1, the PG aggrecan (ACAN), type II collagen (COL2A), the signaling factor sonic hedgehog (SHH), the transcription factor Brachyury [T], the keratins KRT18, KRT19, the carbonic anhydrase CA12, and CD24.^42^

Degenerative NP and AF produce numerous proinflammatory factors including interleukins (IL) −1α, −1β, −2, −4, −6, −8, −10, interferon‐γ, tumor necrosis factor alpha (TNF‐α) and prostaglandin E_2._ 51, 52, 53, 54, 55, 56, 57, 58 Later, chemokine secretion drives specific recruitment and activation of immune cells to the degenerative IVD (eg, CCL5—macrophage/eosinophil recruitment, CCL2—monocyte recruitment, CCL3 and CCL4—macrophage recruitment.^51^, ^59^). Recent work has also implicated mast cell infiltration and proinflammatory action in a similar way as has been observed in other chronic degenerative diseases such as osteoarthritis.^60^, ^61^ Wiet et al demonstrated that mast cells were increased in painful discs and induced inflammatory catabolic responses in NP cells and CEP cells but not in AF cells. This may indicate that the degenerative process provides a means for mast cell infiltration to the NP where they actively promote degeneration, making them and this mechanism a potential therapeutic target.^62^

This environment elicits a range of pathological responses from NP and AF cells including dysregulation of NP‐marker genes such as ACAN and COL2A1 as well as more general processes such as autophagy, senescence, and apoptosis.^6^, ^12^, ^55^, ^63^, ^64^, ^65^ Concurrently, degenerative NP and AF cells increase the expression of matrix degrading enzymes including matrix metalloproteinases (MMPs) −1, −3, −7, −9, −10 −13 and a disintegrin and MMP with thrombospondin motifs (ADAMTS) −1, −4, −5, −9, and −15.^52^, ^55^, ^65^, ^66^, ^67^, ^68^, ^69^, ^70^, ^71^ This further accelerates loss of type II collagen and PG rich ECM and replacement with fibrous, type I collagen scar‐like tissue.

Targeting the cells of the IVD, in particular the NP, to halt the degenerative process and restore healthy ECM production, reverse catabolic processes and reduce inflammatory response is the focus of novel regenerative strategies that have the potential to compliment or replace conventional approaches.

## 3 CURRENT THERAPIES AND DEVELOPMENT OF REGENERATION STRATEGIES

Conservative treatment of degeneration, such as physiotherapy, may indirectly facilitate self‐repair of mildly degenerated IVDs.^72^ These therapies can have positive effects on the lives of patients but do not halt the progression of IVD degeneration and may even mask indicators of further damage requiring more rigorous intervention. When conservative treatments have failed, surgery is used as a final option. As all surgical procedures are invasive and irreversible, they are utilized in less than 2% of symptomatic patients^59^ and are associated with long‐term issues. Where herniation of the IVD has occurred, microdiscectomy remains the gold standard surgical treatment.^73^ Spinal fusion has been extensively used and shown variable success rates of 32% to 98%.^74^ Despite good short‐term outcomes, fusion may result in an accelerated degeneration of the IVDs adjacent to the fusion site due to adjusted load bearing in the spine.^75^, ^76^, ^77^ Alternatively, to take load off the AF a nucleoplasty can be performed, allowing the IVD to return to a normal size and decreasing pressure on symptomatic nerve endings by NP tissue removal. However, this technique is frequently linked to subsequent IVD instability.^78^

In short, the current available surgical treatment options lack the ability to interrupt and correct the degenerative cascade and inflammatory milieu of the degenerative IVD at the necessary cellular and molecular level. Limited use of biologicals in the clinic to enhance surgical procedures such as fusions serve to highlight the potential of these approaches but fall short of providing the regenerative stimulus they may be capable of. The advancement of molecular cell biology and biomaterial science has made the development of effective biologic therapies for IVD regeneration a tangible reality. As our understanding of the biology of the IVD and likewise the pathobiology of degeneration has improved, the number of proposed strategies and molecular targets has grown. These include implantation of cellular and acellular biomaterials, signaling protein‐based strategies such as growth factor delivery and gene therapy. At present, the simplest and most cost‐effective to apply clinically would be local delivery of a bioactive molecule, such as growth factors, morphogens, anticatabolic factors, small molecule inhibitors, cytokines, or chemokines. For example, platelet‐rich plasma, which represents a complex milieu of bioactive factors, has shown promising results both in vitro^79^ and in an in vivo rabbit model where it was injected into degenerated IVDs in gelatin hydrogel microspheres.^80^ However, given the potential variability and unpredictability of using complex mixtures of bioactive factors, research has focused on the identification of single biomolecules with therapeutic potential, with several having have been evaluated in animal models or clinical trials (Table 1). The most commonly investigated candidate molecules are anticatabolic/proanabolic proteins, which aim to restore a healthy balance to the IVD and, in particular, the NP. Anticatabolic factors include small molecule inhibitors of inflammatory signaling, including TNF‐α and IL‐1 receptor antagonists, which have been shown to attenuate the phenotypic changes in IVD cells associated with degeneration.^93^, ^94^ Numerous growth factors have also been investigated, including transforming growth factor (TGF)β3, insulin growth factor (IGF)‐1, and epidermal growth factor‐1.^95^, ^96^, ^97^, ^98^ Most notably, however, a range of members of the bone morphogenetic protein (BMP) family have been investigated due to their roles in skeletal tissue development and repair.

**Table 1:** Biomolecules evaluated for IVD regeneration in clinical trial or preclinical animal models

Drug/material	Product name	Development stage			Outcomes/mode of action		Reference
		Preclinical study	Clinical trial		Available on market		
GDF‐based biological therapies							
GDF‐5	rhGDF5		✓ (Phase 1 and 2a completed) n = 40		Increased disc height. No significant increase in proteoglycan content. No increase NP cell number, increased AF cell number.		NCT011589 Wei et al^24^
GDF‐6		✓ (sheep)			Halted histological evidence of degeneration. Increased NP cell number. Increased NP hydration.		Wei et al^81^
GDF‐6		✓ (rat) (rabbit)			Decreased degeneration‐associated IL‐6, tumor necrosis factor alpha, VEGF, NGF and prostaglandin‐endoperoxide synthase 2 expression. Partial restoration of disc height. Decreased allodynia and evidence supporting nerve cell signaling decrease in rat DRGs in a xenograft NP herniation model.		Miyazaki et al^82^
Other biological therapies							
BMP‐7	Osteogenic protein‐1	✓ (rabbit)	✓ (Phase 1)	✓			An et al^83^
HGF		✓ (rat)			Increased NP water content. Decreased histological score.		Zou et al^84^
IL‐6R mAb	Tocilizumab; Actemra; RoActemra		✓ (Phase 1) n = 31	✓	Decreased calcitonin gene‐related peptide expression in DRG. May be promising analgesic.		Sainoh et al^85^
Link‐N		✓ (rabbit)			Significant increase in aggrecan expression and decrease in proteinase gene expression.		Mwale et al^86^
Hyaluronate hydrogel		✓ (rabbit)			Increased safranin‐O staining.		Nakashima et al^87^
Chondroitin sulfate hydrogel		✓ (rabbit)			Increased safranin‐O staining.		Nakashima et al^87^
Simvastatin	Zocor	✓ (rat)		✓	Increased aggrecan and collagen II expression. Improved histological grades.		Than et al^88^
Lovastatin	Mevacor	✓ (rat)		✓	Increased aggrecan, sox9 and collagen II expression. Decreased collagen I expression. Increased glycosaminoglycan staining.		Hu et al^89^
Glucocorticoid	Hydrocortancyl (Prednisolone)		✓ (Phase 4) n = 137	✓	A single glucocorticoid injection reduces LBP at 1 month but not at 12 months.		NCT00804531 Nguyen et al^90^
Celecoxib	Celebrex	✓ (dog)		✓	No substantial negative effects from gel injection.		Willems et al^91^
Gefitinib (EGFR inhibitor)	Gefitinib	✓ (rat)	✓ (case series)	✓	Slowed histological evidence of IVD degeneration in patients.		Pan et al^92^

Abbreviations: DRG, dorsal root ganglion; GDF, growth differentiation factor; IL, interleukins; IVD, intervertebral disc; NGF, Nerve growth factor; NP, nucleus pulposus; sox9, sex determining region Y‐Box9; VEGF, vascular endothelial growth factor.

Despite its role in osteogenesis, BMP2 has been shown to increase ECM production in rat, bovine, and human IVD cells in vitro without increasing osteogenic marker expression.^99^, ^100^, ^101^, ^102^ Similarly, BMP7 was shown to enhance PG synthesis and cell proliferation in human NP and AF cells,^103^ with others reporting similar results in rat and rabbit IVD cells.^104^, ^105^ However, in human cells, BMP7 was found to be less effective at increasing ECM secretion at similar doses than in rabbit and bovine NP and AF cells.^104^, ^106^ In a recent in vitro and organ culture model, BMP2/7 was also shown to increase glycosaminoglycan (GAG) synthesis in bovine NP cells without increasing osteogenic markers.^107^ However, the majority of these studies failed to investigate NP‐specific or AF‐specific marker genes, often used mixed IVD cell populations and frequently employed only single‐gene analysis to determine osteogenic response. Thus, while they demonstrate the potential of BMP‐based approaches the findings suggest that responses may be cell type specific and be influenced by species, model system, or even local microenvironment, which is further supported by contradictory evidence from in vivo studies. In a rabbit model of degeneration, both BMP2 (adenoviral vector injection) and BMP7 (100‐μg protein injection)‐based therapies demonstrated positive regenerative effects.^108^, ^109^, ^110^ However, a large animal study in goats comparing the efficacy of BMP2, BMP7, and BMP2/7 (1‐5 μg) conjugated to a fibrin/hyaluronic acid carrier showed no evidence of IVD regeneration.^111^ Similarly, in a canine study of spontaneous IVD degeneration, up to 250‐μg BMP7 was injected per IVD but no regeneration was observed.^112^ Such data further suggest that species, cell type, and microenvironment may be important factors in growth factor‐based, particular BMP‐based, regenerative approaches. Concerns also exist with the use of extreme supraphysiological doses of BMPs, for example, up to 12 mg BMP2, which has been linked to adverse effects including heterotopic ossification and increased risk of malignancy^113^, ^114^ meaning a more sophisticated solution combining biomaterial technology or cell‐mediated secretion may be required to enable lower doses and sustained bioactivity to be achieved.

**Figure 1:**
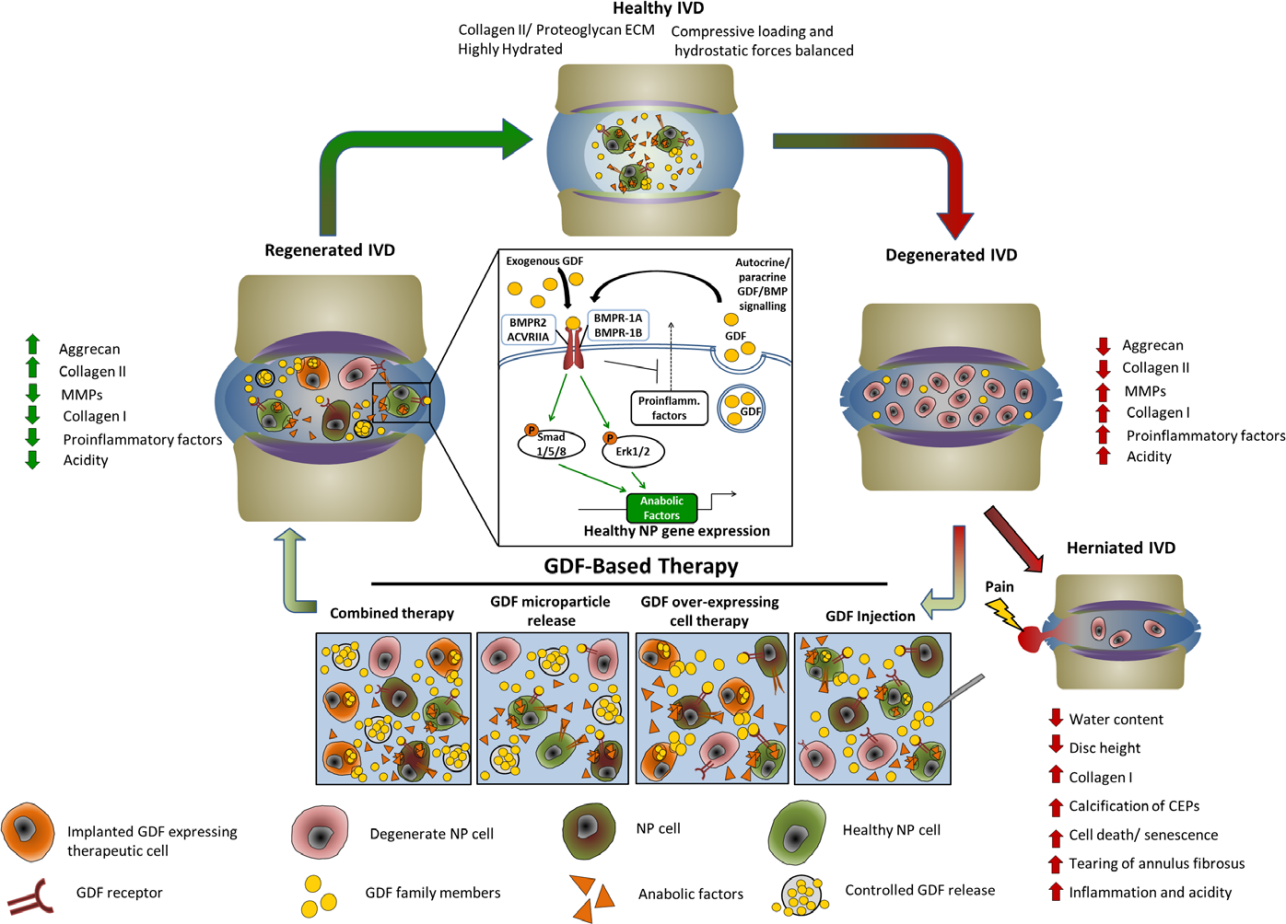
Potential GDF‐based therapeutic cycle for IVD degeneration. A, In the healthy IVD anabolic and catabolic processes are in homeostatic equilibrium. B, In the degenerate IVD cell‐mediated changes, caused by increased inflammatory signaling, shift tissue remodeling processes toward catabolism whilst concomitantly altering ECM production, increasing type I collagen and decreasing proteoglycan production. C, If this process is allowed to proceed, NP herniation can result, causing pain and loss of function. D, Therapeutic intervention involving either injection of GDF, delivery of controlled release GDF microparticles, or combined delivery of GDF with cells has shown promise for readdressing the balance between catabolic and anabolic processes in the degenerate IVD. E, With exogenous GDF delivery the expression of phenotypic and anabolic genes is increased in degenerative NP cells and/or implanted cell populations, thereby restoring disc matrix integrity and function, and reducing pain. F, Summary of GDF signaling pathway through bone morphogenetic protein receptors and smad 1/5/8 which drive anabolic gene expression and inhibit proinflammatory signaling. ECM, extracellular matrix; GDF, growth differentiation factor; IVD, intervertebral disc; MMP, matrix metalloproteinase; NP, nucleus pulposus

In addition to BMPs 2 and 7, other BMP family members have been investigated, with recent data suggesting that members of the GDF family (which includes GDF5 [BMP14, cartilage derived morphogenetic protein 1 (CDMP‐1)], GDF6 [BMP‐13, CDMP‐2], and GDF7 [BMP‐12]) may be ideal molecules for IVD regeneration (Figure 1). Not only do GDFs stimulate anabolic ECM gene expression in IVD cells but have specialized and critical roles in IVD development and homeostasis.

## 4 GDFs IN IVD DEVELOPMENT, HOMEOSTASIS AND POTENTIAL FOR REGENERATION

GDFs were first identified as components of bovine cartilage^115^ and are members of the BMP family. GDF family members share 80% to 86% amino acid homology with each other and around 50% with the wider BMP family.^116^, ^117^, ^118^ BMP family members have been subdivided, on the basis of similarities in protein structure and amino acid sequence, into the BMP2/4 group, BMP‐5/6/7/8 group (OP‐1 [osteogenic protein‐1] group), BMP‐9/10, and the GDF5/6/7 group. Importantly, while most BMP members such as the BMP‐2/4, BMP‐7 (OP‐1), and BMP‐9/10 show strong bone‐inductive activity, GDF5/6/7 induce the formation of cartilage and tendon‐like but not bone‐like tissue.^119^

As with other BMP family members, GDF signaling occurs via heteromeric transmembrane serine‐threonine kinase receptor complexes containing both type I and type II receptor molecules.^120^, ^121^ GDF5 and GDF6 act through the type I receptors bone morphogenetic protein receptor type 1A (BMPR‐1A) and ‐1B and the type II receptors bone morphogenetic protein receptor 2 (BMPR2), activin A receptor type IIA (ACVRIIA), and activin A receptor type IIB (ACVRIIB)^122^, ^123^, ^124^ On receptor binding, GDFs can recruit and activate SMAD1/5/8. Some BMP molecules are also able to signal through non‐Smad kinase cascades; specifically, p38 mitogen‐activated protein kinase (MAPK), Erk1/2 (extracellular related‐signal kinase), and JNK1/2 (c‐JUN N‐terminal kinase) (Figure 1). Interestingly, these pathways are also linked with inflammatory cytokine signaling and mechanical signaling through integrin activation. Recent evidence has demonstrated that GDFs are also able to activate these non‐Smad pathways, in particular Erk1/2.^125^ These noncanonical BMP receptor signaling pathways provide mechanisms linking the anabolic action of factors such as GDF6 and the catabolic proinflammatory signaling, which is a feature of IVD degeneration.

This potential interaction of GDFs and proinflammatory molecules hints at the presence of a natural equilibrium that is dysregulated during degeneration, though this relationship appears to be complex. In vitro, GDF6 increases ECM production in degenerate NP cells,^126^ while in vivo GDF6 treatment prevented ECM degradation in a sheep model.^81^ GDF5 has been shown to directly inhibit the expression of MMP‐13 and ADAMTS4 in human chondrocytes^127^ and expression of other proinflammatory markers such as TNF‐α, IL‐1β, and prostaglandinE2 (PGE2) in murine NP cells.^128^ Meanwhile, IL‐1β and TNF‐α were both found to downregulate GDF5 expression significantly in human AF cells in 3D culture.^129^ In a mouse model, plasma levels of inflammatory factors were downregulated by overexpression of human GDF6.^130^ This indicates an indirect role in the decrease of catabolic enzyme production linked to cellular response to proinflammatory cytokine signaling.

GDF5, 6, and 7 play crucial roles in the development of bones, limb joints, the skull, and axial skeleton^66^ and are expressed in developing cartilage, tendons, and ligaments, as well as in the nervous system.^21^, ^131^, ^132^, ^133^, ^134^ In xenopus and zebrafish embryos, GDF6 is expressed in the ectoderm and acts as an epidermal inducer and a neural inhibitor.^135^, ^136^ In skeletal tissue, GDF family members have a role independent of alkaline phosphatase expression.^122^, ^137^, ^138^ GDF6‐knockout mice display fusions between specific bones in the wrists and ankles, corresponding with major sites of GDF6 expression. Strikingly, in GDF5/6‐knockouts, the vertebral column shows severe lateral curvature, which develops after birth. Alcian blue staining of the IVDs in these mice shows reduced staining indicating lower PG ECM content, despite chondrocyte‐like cells being present.^21^, ^22^ These results suggest that GDF5 and GDF6 are required for normal development and maintenance of the IVD in mice, and correct ECM production by IVD cells. In humans, genomic mutations in the GDF5 gene result in various chondrogenic dysplasias, while polymorphisms in the gene are associated with osteoarthritis.^139^, ^140^, ^141^, ^142^ Similarly, mutations at the GDF6 gene locus on chromosome 8 are known to cause both familial and sporadic cases of Klippel‐Feil syndrome (KFS) leading to similar carpal, tarsal and vertebral fusions as seen in mice GDF5/6 knockouts.^23^ The incomplete nature of these carpal and tarsal fusions in GDF6 knockout mice and in some familial cases of KFS in humans indicates a degree of redundancy in GDF signaling. While variability in spinal fusions between regions of the spine, individuals and species may indicate that GDF6 requirements for complete vertebral segmentation may vary, particularly between different regions of the developing spine.^23^ A recent related report provides evidence that GDF6 is an antiaging factor secreted by young bone marrow‐derived mesenchymal stem cells (MSCs) and its expression can be regulated by miR‐17, a microRNA known to decline with age.^130^ This could have important implications for the progression of IVD degeneration, which is closely associated with the aging process.

During development, cellular secretion of GDFs forms directional morphogenetic gradients.^143^, ^144^, ^145^, ^146^, ^147^ Recently, the expression and localization of GDF6 in developing spinal column of human fetus have been examined.^24^ The strong expression of GDF6 is especially shown throughout cartilaginous region of vertebrae at early developing period (8‐13 weeks of gestation) and in the developing IVDs (between 8 and 19 weeks of gestation) and is diminished from ossification areas. GDF6 expression is localized to the NP and inner AF, but could not be detected in the outer AF by immunohistochemical staining.^24^ In adults, GDF5 and GDF6 are detectable in the inner and outer AF^129^ but are most strongly expressed in the NP.^148^ The precise role of GDF family members in adult IVD homeostasis is unresolved, though there is strong evidence of their importance for anabolic gene expression.

## 5 THE EFFECT OF SUPPLEMENTATION WITH GDFs ON DISC CELLS AND CHONDROCYTES IN VITRO

Aside from driving cell differentiation, GDFs have been shown to have an anabolic effect on IVD cells (Table 2), in particular on NP cells, and chondrocytes in vitro. This indicates a homeostatic or protective role in the IVD, which may be dysregulated during degenerative disc disease. In reported in vitro cultures, GDF5 and GDF6 have similar effects on cells. Recombinant GDF5 stimulation of human NP cells in culture increases aggrecan and type II collagen gene expression and PG production^148^ with similar, dose‐dependent results reported using mouse IVD cells,^151^ and bovine NP and AF cells in culture.^150^ In the latter study, while GDF5 upregulated PG and collagen production in both AF and NP cells, NP cells were the most responsive, especially in terms of PG production (+138% in NP, +24% in AF). In pellet culture, GDF5 was also shown to reduce the expression of catabolic enzyme MMP13 in human chondrocytes through dickkopf 1 inhibition of Wnt signaling.^127^

**Table 2:** Effect of GDF family members on intervertebral disc cells in culture

Cell population	GDF family member/concentration	Culture conditions	Culture duration	Outcomes	Reference
hNP	GDF5 (10 ng/mL)	3D alginate bead culture	Up to 14 Days	i) Significantly increased GAG production. ii) Significantly increased collagen II and aggrecan production.	Le Maitre et al^148^
Mouse GDF5^−/−^ NP	GDF5 (1‐100 ng/mL)	3D alginate bead culture	Up to 9 Days	i) Dose‐dependent upregulation of collagen II and aggrecan expression.	Li et al^149^
Bovine nucleus pulposus	GDF5 (100‐200 ng/mL)	3D alginate bead culture	Up to 21 days	i) 200 ng/mL increased cell proliferation, collagen synthesis and GAG production.	Chujo et al^150^
hNP	GDF6 (200‐400 ng/mL)	3D alginate bead culture	7 days	i) Significantly increased PG production, collagen synthesis and cell migration. 400 ng/mL most effective concentration.	Gulati et al^126^
bAF	GDF6 (adenoviral transfection)	2D	Up to 6 days	i) Significantly increased collagen production, PG secretion and cell proliferation.	Zhang et al^74^
hAF	GDF6	3D alginate bead culture	7 Days	i) Significantly increased PG production, collagen synthesis and cell migration. 400 ng/mL most effective concentration.	Gulati et al^126^

Abbreviations: bAF, bovine annulus fibrosus; GAG, glycosaminoglycan; hAF, human annulus fibrosus; hNP, human nucleus pulposus; PG, proteoglycan.

Through adenoviral‐mediated overexpression studies, Zhang et al compared the effects overexpressing various BMP family molecules on ECM accumulation in bovine AF cells and demonstrated GDF6 overexpression induced one of the most significant PG and collagen production responses.^74^ In 3D alginate bead cultures of human NP and AF cells, GDF6 supplementation increased both PG and collagen production.^126^ Interestingly, at high concentrations (800 ng/mL) GDF6 was also shown to act as a potent chemoattractant for NP cells with similar responses observed to potent chemoattractants, such as fetal calf serum (FCS).^126^, ^152^ This result supports evidence that GDF6 can also act as a chemoattractant to chondrocytes.^153^ In a recent RNA sequencing study investigating regulatory networks controlling ECM synthesis in the human, IVD, GDF6, and GDF5 were identified as important human NP (but not AF) coregulatory gene networks.^154^ GDF6 supplementation also induces aggrecan expression and PG production in articular chondrocytes^155^ and human chondrocyte cell line C28/I2.^153^

Taken together, these results indicate a direct anabolic signaling role for GDF5 and GDF6 in NP and AF cells. Cellular responses and RNAseq data point toward this role being greatest for correct NP cell ECM production. Although AF cells remain responsive to GDF5 and GDF6, their upregulation of PG and collagen production may be a contributory affect, which in vivo supports NP cells to create a functional ECM.

## 6 THE EFFECT OF GDFs ON THE DIFFERENTIATION OF MSCS TOWARD AN NP‐LIKE PHENOTYPE

Numerous growth factors have been investigated as inducers of NP‐like differentiation in MSCs including TGFβ, IGF‐1, fibroblast growth factor 2 (FGF‐2), and platelet derived growth factor (PDGF).^156^, ^157^ However, these studies focus on general chondrogenic markers rather than those markers now associated with specific NP differentiation. Both GDF5 and GDF6 have also been shown to induce the expression of general chondrogenic genes (Sex determining region Y‐Box9 (SOX9); type II collagen; ACAN) and, importantly, to also induce NP‐specific genes (KRT18, KRT19, CA12, Brachyury (T), CD24, HIF1α, Glut‐1 and SHH) in MSCs and adipose‐derived stem cells (ASCs) (Table 3). When GDF5 is added as a media supplement to high‐density cultures of MSCs or ASCs, chondrogenic differentiation is increased; however, several reports also note a significant increase in markers of hypertrophy and ossification including alkaline phosphatase, collagen types I and X, and osteopontin.^151^, ^158^, ^159^, ^160^, ^161^, ^162^, ^163^ These characteristics indicate a progression in expression reflective of chondrogenic hypertrophy toward endochondral ossification, which is undesirable for NP populations. However, limited evidence of a similar response to GDF5 exists in reports investigating NP cells, meaning at present the implications of this observed hypertrophy in stem cell populations for the use of GDF5 for direct application to degenerative discs are unclear and further in vitro and crucially in vivo investigation is required.

**Table 3:** Effect of GDF family members on MSCs in culture

Cell Population	GDF Family Member/ Concentration	Culture Conditions	Culture Duration	Outcomes	Ref.
hMSCs	GDF5	Pellet		i) Significantly increased sGAG and type II collagen production. ii) Increased alkaline phosphatase, types I and X collagen and osteopontin secretion.	Bai et al^151^
hMSCs	GDF5 (50‐500 ng/mL)	Pellet	21 Days	i) Significantly upregulated collagen II. ii) Potential synergistic relationship with TGFβ1 in driving chondrogenic differentiation. iii) No effect on cell proliferation. iv) Concentration of 300 ng/mL found to be most effective.	Coleman et al^158^
Murine Limb bud (E10) + C3H10T1/2	GDF5 (10‐500 ng/mL)	Pellet	Up to 4 Days	i) Significantly increased collagen II and Sox9 expression ii) Significantly increased cell proliferation (after 24 hours). iii) Increased condensation of limb bud cells at 100‐500 ng/mL, stained positively with alcian blue after 3 days culture.	Hatakeyama et al^159^
hMSCs	GDF5 (10‐100 ng/mL)	Woven 3D PLGA scaffolds	12 Days	i) Significantly increased scaffold cellularity. ii) Did not increased collagen production.	Jenner et al^160^
hMSCs and hASCs	GDF5 (10‐1000 ng/mL)	Pellet, 3D collagen I scaffold	14 Days	i) Significantly upregulated collagen II, sox9 and aggrecan expression. Upregulated NP‐specific marker genes (Keratins 8, 18, 19) though not as dramatically. ii) 100 ng/mL most effective. iii) ASCs more responsive than MSCs, some NP‐specific genes not upregulated (CAXII, Brachyury)	Clarke et al^161^
hMSCs	GDF5 (100 ng/mL)	Pellet, followed by agarose mold	7 Days, followed by up to 28 Days cartilage formation	i) Significantly increased aggrecan expression but not collagen II or Sox9. ii) No evidence of hypertrophic marker expression. iii) Evidence of synergy with TGFβ1 to promote chondrogenic differentiation.	Murphy et al^162^
Rabbit ASCs	GDF5 (10‐200 ng/mL)	2D and 3D collagen I sponge	21 Days 2D/ 28 Days 3D	i) In 2D, 100 ng/mL induced collagen II, aggrecan and collagen I expression. 100 to 200 ng/mL most effective. ii) Collagen X expression observed after 21 days. iii) In 3D, GDF5 significantly increased collagen II and aggrecan but also collagen I and X. 3D inducti.on was more potent than 2D	Han et al^163^
hMSCs	GDF5 (100 ng/mL)	3D alginate bead	Up to 18 Days	i) Significantly upregulated aggrecan, collagen II. ii) Promoted high expression of aggrecan in relation to collagen.	Gantenbein‐Ritter et al^164^
hMSCs and hASCs	GDF6 (10‐1000 ng/mL)	Pellet, 3D collagen I scaffold	14 Days	i) Significantly upregulated collagen II, sox9 and aggrecan expression. Upregulated NP‐specific marker genes (Keratins 8, 18, 19, CAXII, Brachyury) in MSCs and ASCs and to greater extent than TGFβ1 GDF5. ii) 100 ng/mL most effective. iii) Increased sGAG secretion and NP‐like proteoglycan: collagen ratio.	Clarke et al^161^
hMSCs	GDF6 (10 ng/mL)	3D collagen I gels, PLA constructs	Up to 14 Days	i) No evidence of collagen X or collagen II response to GDF6 stimulus. Ii) No MMP expression response.	Heckmann et al^165^
C3H10T1/2	GDF6 (adenoviral transfection)	2D	Up to 21 Days	i) Stimulated proliferation. ii) Significantly upregulated collagen II, aggrecan but not collagen X or osteocalcin. iii) 2‐fold increase in GAG secretion vs controls.	Nochi et al^166^

Abbreviations: GDF, growth differentiation factor; hASCs, human adipose‐derived mesenchymal stem cells; hMSCs, human bone marrow‐derived mesenchymal stem cells; PLGA, poly (lactic‐co‐glycolicacid); sGAG, sulphated glycosaminoglycan; sox9, sex determining region Y‐Box9.

In comparative studies, GDF6 promotes greater expression of NP‐marker genes and stimulates greater PG production than TGFβ3 and GDF5 in both human MSCs and ASCs.^161^, ^164^ This increased ratio in PG composition of ECM in comparison to collagenous matrix is a central property of NP tissue and is required for correct functionality. Importantly, reports to date indicate no increase in collagen X production in GDF6 stimulated cultures as seen with other chondrogenic factors.^165^ This finding is supported further in a study by Nochi et al using adenoviral delivery of a GDF6 construct in mesenchymal progenitors (C3H10T1/2 cells). In this study, GDF6 supported chondrogenic differentiation but not terminal differentiation into hypertrophic chondrocytes.^166^ This lack of hypertrophy and progression toward endochondral ossification when using GDF6, coupled with the enhanced expression of NP markers, PG production and the higher aggrecan to type II collagen ratio observed in comparison to GDF5 strongly suggests that GDF6 is the most promising candidate to produce implantable NP cell phenotypes from MSCs or particularly ASCs.

## 7 IN VIVO EFFECTS OF GDFs ON IVD REGENERATION AND DELIVERY METHODS

In preclinical animal models of IVD degeneration treatment with GDF5 and GDF6 have shown promising results. Initial studies investigated delivery of GDF5 and GDF6 through intradiscal injection into models of IVD degeneration. For example, in murine models, IVD recovery after application of static compression was improved with a single GDF5 injection.^167^ After 4 weeks, a significant increase in disc height and cell number was observed in the NP and inner AF, with cells expressing both aggrecan and type II collagen. Similarly, in stab models of degeneration in mouse^168^ and rabbit^150^ delivery of GDF5 improved disc height and histological appearance. Interestingly, GDF5 was found to colocalize with proliferating cells adjacent to the epiphyseal plate in a rabbit model.^169^ Similarly, in an ovine model of annular injury, GDF6 was found to improve defect healing in the AF and improve the hydration and cellularity of the NP.^81^ Recently, Miyazaki and coworkers reported the development and use of a novel rat xenograft radiculopathy model, where rabbit NP tissue was transplanted adjacent to the dorsal root ganglia of nude rats.^82^ The transplanted NP tissue was obtained from annular‐puncture models of degeneration treated either with phosphate buffered saline (PBS) or GDF6 injection. GDF6 injection decreased gene expression, particularly in NP tissue, of proinflammatory factors IL‐6 and TNF‐α (with a trend for decreased IL‐1β) and pain‐associated molecules VEGF, prostaglandin‐endoperoxide synthase 2 (PTGS2), and Nerve growth factor (NGF). More notably, when GDF6‐treated NP was transplanted, transplant‐associated allodynia was significantly reduced. A corresponding decrease in the pain‐related molecules calcium‐binding adaptor molecule‐1 and the nociceptive neuropeptide calcitonin gene‐related peptide in the GDF6‐treated NP associated dorsal root ganglions (DRGs) was also observed.

One potentially interesting role for GDF6 is as a chemotactic factor for stem/progenitor cell populations adjacent or resident in the NP. This could be relevant either as a homeostatic tissue maintenance process or during repair. Other related BMP family members, such as BMP2, have been demonstrated to act as chemotactic signals for mesenchymal progenitor cells^170^ and as mentioned above GDF6 acts as a chemoattractant to NP cells in vitro.^126^ Populations of IVD progenitors have been identified in inner AF region of rabbit discs,^171^, ^172^, ^173^ and in both human nondegenerate scoliotic AF^174^ and degenerate AF tissues.^175^ This hypothetical mechanism is also supported by the animal studies referred to above, where cells were observed to migrate from the endplates toward the AF and NP injury site. However, the identity and characteristics of these cells, including their secretory profile, remains to be defined.

Injection of supraphysiological doses of growth factors is unlikely to be effective for the long‐term treatment of degenerative IVD disease. This is due to the short half‐life of these growth factors in vivo, while the large doses delivered raise safety concerns due to off‐target effects. Furthermore, repeated injection of the IVD may compromise its mechanical integrity and has been shown to cause an inflammatory reaction at the injection site.^167^ Thus, the development of alternative GDF delivery methods is desirable. These could include delivery of the GDF gene through adenoviral or plasmid vectors, delivery of siRNAs to block pathways that may downregulate GDF signaling, miRNA delivery/ inhibition or gene editing techniques such as clustered regularly inter spaced short palindromic repeats (CRISPR). Other methods might rely on the controlled delivery of GDF protein, such as microsphere encapsulation and tethering/presentation of GDF coupled with a biomaterial. These options could be used either to manipulate the endogenous cell populations or be coupled with a cellular implant, such as MSCs or ASCs (Figure 1).

In a proof of principle mouse annulus needle puncture model of IVD degeneration, Liang et al injected an adenoviral vector carrying the GDF5 gene into lumbar discs.^168^ The study demonstrated that the GDF5 gene was successfully expressed and active GDF5 produced, which leads to significant restoration of disc height, histology, and improved disc hydration as assessed through magnetic resonance imaging.^168^ Adenoviral transduction of murine mesenchymal progenitors was also shown to be feasible for the expression of human GDF6, where GDF6 induced expression of chondrogenic genes and increased PG production without expression of hypertrophic genes.^166^

Nonviral transfection of GDFs into NP cells or MSCs is perhaps a more attractive option through reduction of safety concerns associated with the use of viral vectors. Nonviral transfection of GDF5 into electroporated MSCs was shown to result in efficient GDF5 expression up to 21 days in culture, upregulate NP‐gene expression and partially recover the GAG/DNA ratio in a bovine IVD degeneration organ culture model.^176^ In principle, nonviral transfection of primary IVD cells has recently been demonstrated by May et al using the Neon Transfection System to achieve transfections efficiencies ≥47%.^177^

Alternatively, a controlled GDF delivery system would allow prolonged delivery of GDFs to the degenerate IVD through prevention of degradation, allow the use of lower GDF concentrations, and localize delivery. Encapsulation in polymer microspheres is a commonly used method to control release of growth factors. In a rat needle puncture disc degeneration model, GDF5 encapsulated in poly (lactic‐co‐glycolicacid) (PLGA) microspheres and delivery was sustained over 42 days, which resulted in GAG increases and restoration of disc height.^178^

These studies indicate an anabolic role for GDF5 and GDF6 in the IVD. Although as mentioned above, GDF5 and GDF6 are present in the degenerate IVD, it is clear that GDF effector signaling is dysregulated, resulting in decreased anabolic gene and protein expression. In these in vivo studies providing an excess of GDF6 can recover this anabolic expression, suggesting a rebalancing of an equilibrium between inflammatory catabolic signaling and GDFs. Indeed, evidence of noncanonical GDF signal transduction supports the direct interaction of these processes. A successful GDF treatment therefore may involve a GDF delivery or overexpression system in concert with an inhibitor of inflammatory response. Delivery period and dosage must be determined through in vivo experiments and perhaps can be tailored with patient specificity through more accurate diagnostic processes. Similarly, whether a cellular or acellular approach is required is dependent on patient‐specific degeneration phase. The stimulation of endogenous cell populations to either increase GDF production or delivery of GDF may only be possible if degeneration remains in the early stages. In this way, IVD regeneration would benefit greatly from a stratified approach to treatment design. Fortunately, hydrogels in combination with microparticle controlled delivery, for example, with or without cells, lend themselves to this kind of modular adaptation for treatment. GDFs, in particular GDF6 with its highly NP‐specific responses and lack of hypertrophic gene expression, are exciting prospects for IVD regeneration.

## 8 CONCLUSION AND FUTURE DIRECTION

Small molecule therapy, especially in combination with cell implantation, holds promise for the treatment of degenerative disc diseases. The link between ECM composition and tissue functionality in the NP means that it is critical to select the correct bioactive molecule, or combination of molecules. GDF family members have shown great promise both as direct anabolic factors when delivered to NP cells and as NP‐specific differentiation‐stimuli when delivered to MSCs or ASCs. In either case, GDF6 has been shown to increase GAG production relative to type II collagen and upregulate healthy NP‐specific marker gene expression, and critically, to do so to a greater extent than other previously defined chondrogenic factors. Future work will focus on the delivery and controlled release of GDF family members to the disc. The combination of GDF with cell therapy should also be a focus going forward, with work underway to determine optimal cell populations for therapy. In vivo data hinting at a role for GDF6 in modulation of degeneration‐associated pain and the mobilization and localisation of tissue resident stem cells during healing of artificially induced degeneration is exciting. The precise nature of these interactions should be explored in more detail but perhaps they offer a potential avenue for effective cell‐free delivery of GDF family members. Given the proinflammatory environment of the degenerative IVD and the convergence of noncanonical GDF and cytokine signaling on kinase cascades, further research is needed to determine the nature of this interaction, which will likely lead to the identification of novel therapeutic targets that may optimize GDF therapy.

## CONFLICTS OF INTEREST

A.D. acts as educational consultant to Nuvasive. Nuvasive provides an unrestricted donation to University of New South Wales for spinal fellow education and research. A.D. is also a patent holder on biologic therapies for spinal disease and may benefit if ever the patents are licensed or commercialized.
